# Implications of new-onset atrial fibrillation on in-hospital and long-term prognosis of patients with acute myocardial infarction: A report from the CBD bank study

**DOI:** 10.3389/fcvm.2022.979546

**Published:** 2022-10-28

**Authors:** Wang-Yang Yang, Gregory Y. H. Lip, Zhi-Jun Sun, Hui Peng, Ameenathul M. Fawzy, Hong-Wei Li, The Cardiovascular Center Beijing Friendship Hospital Database Bank (CBD Bank) study group

**Affiliations:** ^1^Department of Cardiology, Cardiovascular Center, Beijing Friendship Hospital, Capital Medical University, Beijing, China; ^2^Liverpool Centre for Cardiovascular Science at University of Liverpool, Liverpool John Moores University and Liverpool Heart & Chest Hospital, Liverpool, United Kingdom; ^3^Department of Clinical Medicine, Aalborg University, Aalborg, Denmark

**Keywords:** acute myocardial infarction, new-onset atrial fibrillation, in-hospital prognosis, long-term prognosis, real world study

## Abstract

**Background:**

An increase in the incidence of atrial fibrillation (AF) during the acute phase of myocardial infarction (AMI) has been observed. But it is still unclear whether the implications of new-onset AF on in-hospital and long-term prognosis are of similar magnitude.

**Methods:**

Using data from the CBD Bank study, 3,824 consecutive AMI patients, without prior AF, were analyzed. During the index hospitalization, all patients were monitored by continuous cardiac monitoring, twice daily performed 12- or 18-lead ECGs and timely ECG checks when cardiac symptoms occurred. Follow-up visits were routinely scheduled after discharge. Primary outcomes were all-cause death and cardiovascular death occurring during hospitalization and long-term follow-up. Secondary outcome was MACEs during hospitalization.

**Results:**

During the median hospital stay of 9.0 (7.0, 11.0) days, new-onset AF was documented in 133 (3.48%) patients; 95 (71.43%) patients had AF attacks within 3 days following AMI. Independent risk factors associated with new-onset AF were older age, larger left atrial diameter, higher level of NT-proBNP, and primary PCI. New-onset AF was found to be significantly associated with in-hospital all-cause death (OR 4.33, 95%CI: 2.37-7.89, *P* < 0.001), cardiovascular death (OR 4.10, 95%CI: 2.18-7.73, *P* < 0.001), and MACEs (OR 2.51, 95%CI: 1.46-4.33, *P* = 0.001). A total of 112 new-onset AF and 3,338 non-AF patients were followed up for 1,090 (365, 1,694) days after discharge. There was no significant association between new-onset AF and long-term all-cause death (HR 1.21, 95%CI: 0.77-1.92, *P* = 0.406) or cardiovascular death (HR 1.09, 95%CI: 0.61-1.97, *P* = 0.764).

**Conclusion:**

New-onset AF following AMI is strongly associated with an increased risk of adverse in-hospital prognosis, but it does not affect prognosis in those who survive until hospital discharge.

## What’s new?

• The present study analyzed data from a large hospital-based prospective cohort study, in order to assess the incidence of new-onset AF during the initial hospitalization for AMI on current aggressive revascularization therapy and intensive pharmacotherapy, investigate the associated risk factors, and determine whether the implications of new-onset AF on in-hospital and long-term prognosis are of similar magnitude.
• The incidence of new-onset AF in patients with AMI was higher than that in the general population, with an incidence of 3.48% in our study.
• New-onset AF occurring concurrently with or after AMI during the index hospitalization is strongly associated with an increased risk of adverse in-hospital prognosis; but it does not affect prognosis in those who survive until hospital discharge.
• Older age, higher levels of NT-proBNP, larger left atrium diameter, and primary PCI were identified as risk factors for new-onset AF following AMI.

## Introduction

Atrial fibrillation (AF) affects about 1% of the general population, with a higher incidence in specific clinical subsets, such as the elderly and those with hypertension, heart failure, and coronary artery disease. A marked increase in the incidence of AF during the acute phase of myocardial infarction (AMI) has been observed (1–3). Up to 9% of patients with ST-segment elevation myocardial infarction (STEMI) develop AF during or immediately after percutaneous coronary intervention (PCI) (4). Importantly, new-onset AF following AMI seems to be associated with clinical outcomes (1, 5). However, the prognostic implications remain controversial, especially whether the implications of new-onset AF on in-hospital and long-term prognosis are of similar magnitude. The epidemiology of AF following AMI may also vary depending on current aggressive revascularization therapy and intensive pharmacotherapy (2, 6). As the existing data is mostly derived from clinical trials or registry studies, their applicability to real-world clinical practice is uncertain. Also, there remains a lack of evidence regarding the influence of new-onset AF following AMI in the Asian population.

The Cardiovascular Center Beijing Friendship Hospital Database Bank (CBD Bank) study (7) is a hospital-based prospective cohort study, consisting of patients hospitalized for cardiovascular disease, providing an opportunity to follow the real-world epidemiology and management of patients with AMI. Using data from the CBD Bank study, we aimed to assess the incidence of new-onset AF during the initial hospitalization for AMI, investigate the associated risk factors, and determine the implications of new-onset AF on in-hospital and long-term prognosis.

## Materials and methods

### Study population

Based on the CBD Bank study, consecutive adult patients with AMI were enrolled in our study, in the absence of exclusion criteria which were as follows: (i) Patients with previously diagnosed AF; (ii) No baseline echocardiography; (iii) age ≥90 years old or severe concomitant disease with life expectancy of less than one year. The data collection process was approved by the Institutional Review Board of Beijing Friendship Hospital affiliated to Capital Medical University and was in accordance with the Declaration of Helsinki.

In our study, AMI was defined as detection of serum cardiac markers elevation with at least one value above the 99th percentile upper reference limit with clinical evidence of acute myocardial ischemia (8). And they were classified as ST-segment elevation myocardial infarction (STEMI), or non-ST-segment elevation myocardial infarction (NSTEMI) according to the ECGs.

All the coronary angiography procedures were performed at the angiographic core laboratory in Beijing Friendship Hospital. Revascularization strategies during the index hospitalization were at the discretion of cardiologists following guideline recommendations (9). Extenuating circumstances exist in the clinical scenarios such as but not limited to unwillingness to consider revascularization, comorbidities likely to markedly increase procedural risk, or technical reasons making revascularization infeasible.

Regardless of whether or not primary PCI was performed, all patients with AMI were firstly admitted into cardiac intensive care unit (CCU) for about one week, with continuous cardiac monitoring, twice daily performed 12- or 18-lead ECGs, and timely ECG checks when cardiac discomfort occurred. Following this, patients were stepped down from CCU to the general ward, where ECGs were performed based on symptoms.

### Data collections and definitions

Baseline clinical data such as patient’s demographics, medical history, laboratory test results, echocardiography, and angiography evaluation results were captured from the electrical medical record system with a consistent search strategy and randomly validated through manual review. The level of myocardial enzymes and N-terminal pro-B-type natriuretic peptide (NT-proBNP) were monitored during hospitalization. TnI_max_ represents the peak serum concentration of cardiac troponin I (TnI). NT-proBNP_max_ signifies the peak serum concentration of NT-proBNP. Other laboratory test results were based on the first measurement, typically on admission.

New-onset AF was defined as newly detected AF occurring concurrent with or after AMI during the index hospital stay, which was detected by discharge diagnosis of new AF and verified by re-check of ECG and medical records. In our study, AF was defined as the episode of AF or atrial flutter that lasted at least 30s captured on ECG or cardiac monitor. AF burden was regarded as the duration of AF as a proportion of the total length of hospital stay. Non-AF was defined as no diagnosis of AF throughout the index hospitalization. The study cohort was divided into patients with new-onset AF and patients with non-AF, accordingly. The treatment of patients with new-onset AF, including anticoagulation and antiarrhythmic therapy, was at the discretion of the attending physicians. In general, beta-blocking agents were recommended for rate control. Amiodarone or electrical cardioversion was used for heart rhythm control, especially in hemodynamically compromised patients. Low molecular weight heparin (LMWH) or oral anticoagulants (OAC, warfarin or non-vitamin K oral anticoagulant) were encouraged in high thromboembolic risk patients during hospitalization. Whether OAC in additional to dual antiplatelet drugs was prescribed at discharge was determined by weighing the bleeding and thromboembolism risks (10, 11).

### Follow-up and clinical outcomes

Patients who survived hospitalization and signed informed consent were followed up from discharge. The follow-up visits were routinely scheduled at 1, 3, 6, 12 months, and annually thereafter, by trained staff at outpatient clinics or via telephone interviews.

The primary outcomes were all-cause death and cardiovascular death occurring during hospitalization and long-term follow-up. Secondary outcomes were the major adverse cardiac events (MACEs) during the index hospitalization, including cardiovascular death, revascularization, malignant arrhythmia, recurrent myocardial infarction, ischemic stroke, and cerebral hemorrhage. All-cause death was defined as death from all causes, while cardiovascular death was specific to that which occurred due to AMI, stroke, sudden death, and other cardiovascular comorbidities (12). Deaths without a clear non-cardiovascular cause were also classified as cardiovascular deaths. Patients were followed up from the initial discharge day to the date of death, loss of follow-up, or the end of the observational period (the censoring date of 31 June 2020), whichever came first. Events were adjudicated by the trained investigators by telephone interview and comprehensive review of the patient’s inpatient and outpatient medical records. Controversial events were adjudicated by an independent clinical events committee.

### Statistical analysis

Continuous variables are presented as means ± standard deviations (SD) or as median (InterQuartile Range (IQR), i.e., quartile1-quartile3), and *T*-tests or Wilcoxon rank-sum tests are used accordingly. Categorical variables are expressed as numbers (percentages) and compared using Chi-squared (χ^2^) test. Univariable and multivariable logistic regression models were used to identify risk factors independently associated with new-onset AF. The cut-off value in transforming continuous variables into binary variables was based on the baseline median value or the value of clinical significance. Covariates included in the multivariable model were those which were significantly associated with new-onset AF in the univariable analysis and previously published studies. Hence, the selected covariates were age ≥75 years, male sex, STEMI, history of hypertension, diabetes mellitus, heart failure, NT-proBNP_max_, TnI_max_, estimated glomerular filtration rate (eGFR), high sensitivity C-reactive protein (hCRP), left atrium diameter (LAd), left ventricular ejection fraction (LVEF), Primary PCI, and stenosis of the left circumflex (LCX), right coronary artery (RCA) or left main coronary artery (LM). The incidence of long-term clinical outcome events was calculated by dividing numbers of events by person-years at risk, with the 95% confidence interval (CI) estimated using a Poisson model. Cumulative survival free from long-term outcome events was presented with a Kaplan-Meier curve, and the difference was compared using the log-rank test. Logistic regression analysis was performed to assess the implication of new-onset AF on in-hospital outcomes; cox proportional hazard analysis was used to examine the association between new-onset AF and long-term outcomes, all adjusted by age ≥75 years, male sex, history of hypertension, diabetes mellitus, chronic kidney disease, prior stroke, NT-proBNP_max_ ≥ 2,000 pg/ml, TnI_max_ ≥ 5 ng/ml, LAd ≥ 40 mm, LVEF ≥ 50%, and Primary PCI. Analyses were performed using SPSS Statistics version 22.0 (IBM) and SAS software version 9.4 (SAS Institute, Cary, NC, USA), and a two-sided *P*-value <0.05 was considered statistically significant.

## Results

**FIGURE 1:**
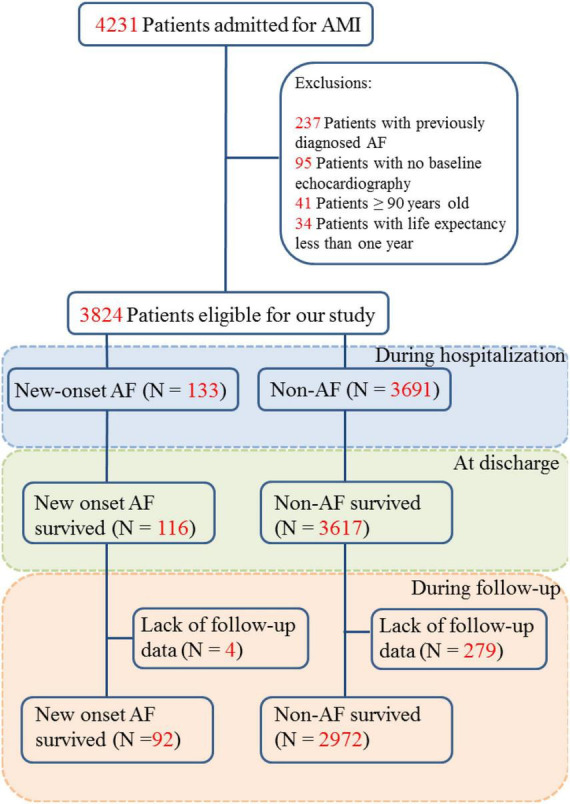
Flow chart of patients included in the study.

As shown in the flow chart (Figure-1), 4231 patients were hospitalized for incident AMI between December 2012 and December 2019. After exclusion, 3824 patients were eligible for our study. New-onset AF was documented in 133 (3.48%) patients during a median hospital stay of 9.0 (7.0, 11.0) days. Of these, 56 (42.11%) patients had documented AF on admission, another 39 (29.32%) patients had AF attacks within 3 days following AMI. The mean new AF onset time was 1.0 (0.5, 4.0) days after AMI. The fastest ventricular rate recorded in AF was 115 ± 31.5 bpm. The average duration of AF episodes was 1.0 (0.5, 3.0) days. Thus, the AF burden was calculated to be 11.10 (5.45, 31.65)%. During hospitalization, 117 (87.97%) patients with new-onset AF accepted anticoagulants in additional to dual antiplatelet drugs. Eleven (8.27%) patients were prescribed with OAC combined with dual antiplatelet drugs at discharge. During hospitalization, 76 (51.14%) patients converted to sinus rhythm spontaneously, 49 (36.84%) were converted by intravenous amiodarone, one underwent electrical cardioversion, and 7 (5.26%) only accepted rate control.

Baseline characteristics of the study cohort stratified by the presence of new-onset AF were summarized in Table 1. Patients with new-onset AF were older, more likely to be female, and more likely to have presented with a STEMI. They also had longer hospital stays, a higher prevalence of heart failure, higher levels of NT-proBNP_max_, TnI_max_, and hCRP, larger LAd, and were more likely to have undergone primary PCI, while having a lower eGFR and LVEF compared with non-AF patients.

**TABLE 1:** Baseline characteristics.

	New-onset AF (*n* = 133)	Non-AF (*n* = 3691)	*P-value*
Age, years, mean ± SD	72.02 ± 10.77	63.72 ± 12.21	<0.001
Age ≥ 75y, *n* (%)	66 (49.6)	841 (22.8)	<0.001
Male sex, *n* (%)	82 (61.7)	2764 (74.9)	0.001
STEMI, *n* (%)	79 (59.4)	1828 (49.5)	0.025
BMI ≥ 28kg/m^2^, *n* (%)	27 (20.3)	823 (22.3)	0.586
Systolic blood pressure, mmHg, mean ± SD	125.98 ± 25.33	129.06 ± 22.12	0.169
Duration of hospitalization, days, median (IQR)	12.0 (9.0, 17.0)	9.0 (7.0, 11.0)	<0.001
**Comorbidities**			
Hypertension, *n* (%)	91 (68.4)	2,358 (63.9)	0.284
Diabetes mellitus, *n* (%)	51 (38.3)	1,211 (32.8)	0.182
Dyslipidemia, *n* (%)	49 (36.8)	1,654 (44.8)	0.069
CAD, *n* (%)	43 (32.3)	1,153 (31.2)	0.789
Prior MI, *n* (%)	15 (11.3)	434 (11.8)	0.866
Prior PCI, *n* (%)	14 (10.5)	391 (10.6)	0.980
Heart failure, *n* (%)	4 (3.0)	29 (0.8)	0.025
Peripheral artery disease, *n* (%)	8 (6.0)	192 (5.2)	0.679
Chronic kidney disease, *n* (%)	12 (9.0)	206 (5.6)	0.093
Prior ischemic stroke, *n* (%)	24 (18.0)	594 (16.1)	0.548
**Laboratory test**			
NT-proBNP_max_*, pg/ml, median (IQR)	7,097.0 (2,241.0, 13,126.0)	1,537.0 (564.0, 4,106.5)	<0.001
TnI_max_*, ng/ml, median (IQR)	10.1 (1.0, 30.0)	4.7 (1.0, 16.5)	0.009
eGFR, ml/min/1.73 m^2^, mean ± SD	67.08 ± 28.15	81.32 ± 24.93	<0.001
eGFR ≤ 30 ml/min/1.73 m^2^, *n* (%)	12 (9.0)	142 (3.8)	0.003
HbA1c,%, mean ± SD	6.58 ± 1.21	6.56 ± 1.56	0.852
hCRP, mg/L, median (IQR)	9.0 (3.0, 24.9)	6.0 (2.1, 15.1)	0.001
TSH, uIU/ml, median (IQR)	1.0 (0.6, 1.8)	1.2 (0.7, 2.0)	0.106
**Echocardiography parameters**			
LAd, mm, mean ± SD	40.36 ± 5.77	37.47 ± 4.51	<0.001
LAd ≥ 40 mm, *n* (%)	74 (55.6)	1,111 (30.1)	<0.001
LVEF,%, mean ± SD	54.81 ± 9.46	58.48 ± 10.35	<0.001
LVEF ≤ 50%, *n* (%)	34 (25.6)	688 (18.6)	0.045
EDD, mm, mean ± SD	52.62 ± 6.62	52.36 ± 5.75	0.616
**Primary PCI^#^**	56 (42.1)	1104 (29.9)	0.003

*Serum peak concentration during hospitalization. ^#^Patients underwent PCI within 12 h after symptom onset. AF, atrial fibrillation; BMI, body mass index; CABG, coronary artery bypass surgery; CAD, coronary artery disease; CK-MB, creatine kinase-myocardial band isoenzyme; eGFR, estimated glomerular filtration rate; ESR, erythrocyte sedimentation rate; HbA1c, hemoglobin A1c; hCRP, high sensitivity C-reactive protein; LAd, left atrium diameter; LVEF, left ventricular ejection fraction; MI, myocardial infarction; MYO, myoglobin; NT-proBNP, N-terminal pro-B-type natriuretic peptide; PCI, percutaneous coronary intervention; SD, standard deviations; STEMI, ST-segment elevation myocardial infarction; Scr, serum creatinine; TnI, troponin I; TSH, thyroid-stimulating hormone.

A total of 3,301 patients had coronary angiography examination during the index hospitalization, 56 (42.1%) new-onset AF patients, and 1,104 (29.9%) non-AF patients underwent primary PCI. Other patients received conservative treatment with antiplatelet and/or anticoagulant drugs. Angiographic data pertaining to the coronary artery lesions are presented in Table 2, where significant differences were observed in LM, LCX, and RCA stenosis between new-onset AF and non-AF patients.

**TABLE 2:** Angiographic parameters (*N* = 3301).

	New AF (*n* = 108)	No AF (*n* = 3193)	*P-value*
LM stenosis > 50%, n (%)	14 (13.0)	210 (6.6)	0.009
**Artery with stenosis > 75%, n (%)**			
LAD	77 (71.3)	2,103 (65.9)	0.241
LCX	56 (51.9)	1,346 (42.2)	0.045
RCA	65 (60.2)	1,432 (44.8)	0.002

LM, left main coronary artery; LAD, left anterior descending artery; LCX, left circumflex; RCA, right coronary artery.

### Risk factors for new-onset atrial fibrillation

Univariable and multivariable analyses for risk factors associated with new-onset AF following AMI are summarized in Table 3. Univariable analysis revealed that age ≥75 years, male sex, STEMI, history of heart failure, NT-proBNP_max_ ≥ 2,000 pg/ml, TnI_max_ ≥ 5 ng/ml, eGFR ≤ 30 ml/min/1.73m2, hCRP ≥ 6 mg/L, LAd ≥ 40 mm, LVEF ≤ 50%, primary PCI, LM stenosis > 50%, LCX stenosis ≥75%, and RCA stenosis ≥75% was associated with higher risk of new-onset AF, while TSH ≥ 1 uIU/ml was associated with lower risk of that (all *p* < 0.05).

**TABLE 3:** Risk factors of New-onset atrial fibrillation after acute myocardial infarction.

			Multivariate	
	Univariate		Model (N′ = 3301)	
	OR (95%CI)	*P* value	OR (95%CI)	*P-value*
Age ≥ 75 y	3.34 (2.36, 4.73)	<0.001	2.09 (1.34, 3.26)	0.001
Male sex	0.54 (0.38, 0.77)	0.001	0.93 (0.59, 1.47)	0.757
BMI ≥ 28 kg/m^2^	0.89 (0.58, 1.36)	0.587		
STEMI	1.49 (1.05, 2.12)	0.026	0.99 (0.58, 1.69)	0.957
Systolic blood pressure	0.99 (0.99, 1.00)	0.117		
Hypertension	1.23 (0.85, 1.78)	0.285	0.98 (0.63, 1.52)	0.924
Diabetes mellitus	1.27 (0.89, 1.82)	0.183	1.02 (0.67, 1.56)	0.920
Dyslipidemia	0.72 (0.50, 1.03)	0.070		
Prior CAD	1.05 (0.73, 1.52)	0.789		
Prior MI	0.95 (0.55, 1.65)	0.866		
Prior PCI	0.99 (0.57, 1.75)	0.980		
Prior CABG	1.53 (0.47, 4.94)	0.481		
Heart failure	3.92 (1.36, 11.30)	0.012	1.38 (0.17, 11.54)	0.767
Peripheral artery disease	1.17 (0.56, 2.42)	0.679		
Chronic kidney disease	1.68 (0.91, 3.09)	0.096		
Prior ischemic stroke	1.15 (0.73, 1.80)	0.548		
NT-proBNP_max_ ≥ 2,000 pg/ml	5.68 (3.72, 8.66)	<0.001	3.63 (2.18, 6.04)	<0.001
TnI_max_ ≥ 5 ng/ml	1.93 (1.36, 2.73)	<0.001	1.35 (0.82, 2.20)	0.236
HbA1c	1.01 (0.90, 1.13)	0.883		
eGFR ≤ 30 ml/min/1.73 m^2^	2.48 (1.34, 4.59)	0.004	1.89 (0.84, 4.24)	0.124
hCRP ≥ 6 mg/L	1.49 (1.05, 2.11)	0.025	1.03 (0.69, 1.55)	0.883
TSH ≥ 1 uIU/ml	0.69 (0.49, 0.98)	0.037	0.73 (0.49, 1.11)	0.138
LAd ≥ 40 mm	2.91 (2.05, 4.13)	<0.001	2.37 (1.56, 3.59)	<0.001
LVEF ≤ 50%	1.50 (1.01, 2.23)	0.046	0.74 (0.46, 1.20)	0.225
Primary PCI	1.70 (1.12, 2.42)	0.003	1.89 (1.11, 3.19)	0.018
LM stenosis > 50%	2.12 (1.19, 3.77)	0.011	1.62 (0.87, 3.01)	0.128
LAD stenosis ≥ 75%	1.29 (0.84, 1.97)	0.242		
LCX stenosis ≥75%	1.48 (1.01, 2.17)	0.046	1.21 (0.80, 1.82)	0.368
RCA stenosis ≥75%	1.86 (1.26, 2.75)	0.002	1.30 (0.85, 1.97)	0.225

Abbreviations as in Table 1.

In the multivariable analysis, independent factors associated with new-onset AF were age ≥75 years (OR 2.09, 95%CI: 1.34–3.26, *P* = 0.001), NT-proBNP ≥ 2,000 pg/ml (OR 3.63, 95%CI: 2.18–6.04, *P* < 0.001), LAd ≥ 40 mm (OR 2.37, 95%CI: 1.56–3.59, *P* < 0.001), and primary PCI (OR 1.89, 95%CI: 1.11-3.19, *P* = 0.018).

### New-onset atrial fibrillation and in-hospital prognosis

During the index hospitalization, 17 (12.8%) new-onset AF patients died due to all causes, of which 15 (11.3%) were cardiovascular deaths. A total of 18 (13.5%) MACEs occurred in this group. For non-AF patients, 74 (2.0%) all-cause deaths, 65 (1.8%) cardiovascular deaths, and 168 (4.6%) MACEs occurred. Specifically, one (0.75%) patient had stroke in the new-onset AF group; 15 (0.41%) patients had stroke and 7 (0.19%) patients had cerebral hemorrhage in the non-AF group. After multivariable adjustment, new-onset AF was significantly associated with in-hospital all-cause death (OR 4.33, 95%CI: 2.37-7.89, *P* < 0.001), cardiovascular death (OR 4.10, 95%CI: 2.18-7.73, *P* < 0.001), and MACEs (OR 2.51, 95%CI: 1.46-4.33, *P* = 0.001).

### New-onset atrial fibrillation and long-term prognosis

After excluding the in-hospital deaths and a further 283 (7.58%) patients who did not have follow-up data, 3,450 patients were included in the long-term follow-up analysis: 112 with new-onset AF and 3,338 non-AF patients.

**FIGURE 2:**
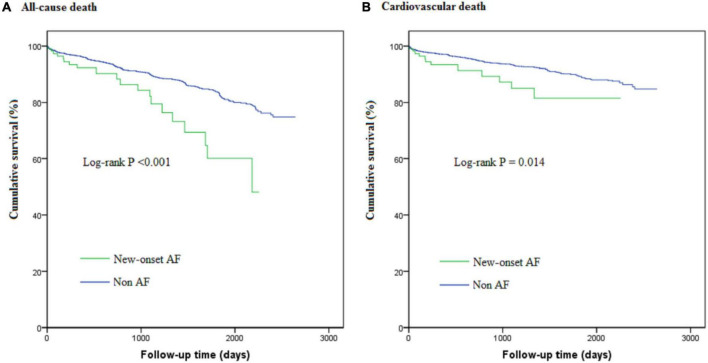
The Kaplan-Meier curves for cumulative survival free from all-cause death **(A)** and cardiovascular death **(B)**.

**FIGURE 3:**
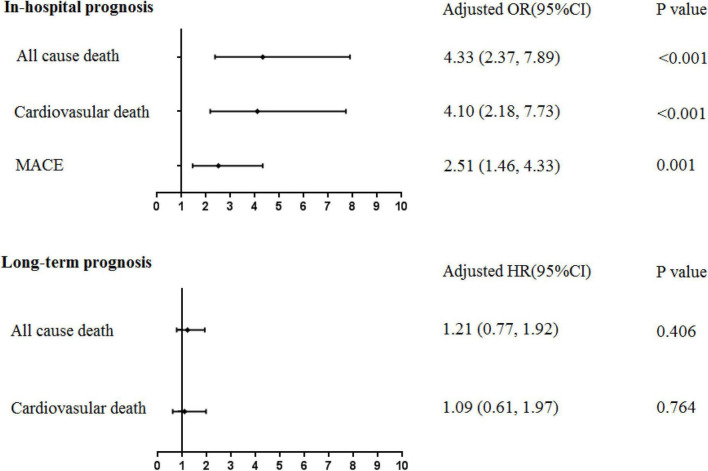
The association between new-onset AF following AMI and in-hospital/long-term prognosis. MACE, major adverse cardiovascular events. Multivariable regression analysis, adjusted by age ≥75 years, male sex, history of hypertension, diabetes mellitus, chronic kidney disease, prior stroke, NT-proBNP_max_ ≥ 2,000 pg/ml, TnI_max_ ≥ 5 ng/ml, LAd ≥ 40 mm, LVEF ≥ 50%, and Primary PCI.

After a median follow-up period of 1,090 (365, 1,694) days, all-cause death occurred in 20 new-onset AF patients and 366 non-AF patients; corresponding rates were 8.54 (95%CI: 5.51–13.24) vs. 3.78 (95%CI: 3.41–4.19) per 100 person-years, respectively. Cardiovascular death occurred in 12 new-onset AF patients and 230 non-AF patients; corresponding rates were 5.12 (95%CI: 2.91-9.02) vs. 2.38 (95%CI: 2.09-2.70) per 100 person-years, respectively. The cumulative survival free from all-cause death and cardiovascular death stratified by new-onset AF is shown in the Kaplan-Meier curve in Figure 2. Although patients with new-onset AF seemed to have higher long-term death rate, such an association was attenuated after multivariable adjustment. Thus, there was no significant association between new-onset AF and long-term all-cause death (HR 1.21, 95%CI: 0.77–1.92, *P* = 0.406) or cardiovascular death (HR 1.09, 95%CI: 0.61–1.97, *P* = 0.764). The implications of new-onset AF on the in-hospital and long-term prognosis were summarized in Figure 3.

## Discussion

The present study analyzed hospital-based data from a large cohort of AMI patients. The incidence, risk factors, and prognostic implication of new-onset AF in patients with AMI were examined and the major findings were as follows: (i) The incidence of new-onset AF in patients with AMI was higher than that in the general population, with an incidence of 3.48% in our study; (ii) Older age, higher levels of NT-proBNP, larger LAd, and primary PCI were identified as risk factors for new-onset AF following AMI; and (iii) New-onset AF was strongly associated with a higher risk of adverse in-hospital prognosis, including all-cause death, cardiovascular death, and MACE, but this association was not observed for long-term mortality. Our findings suggest that new-onset AF needs to be further evaluated particularly during the initial hospitalization for AMI patients.

The incidence of new-onset AF in AMI settings varies in different studies, ranging from 2 to 22% (3, 5, 10, 13, 14). As a hospital-based study, the incidence of new-onset AF following AMI in our population is consistent with prior reports. Apart from the heterogeneity of different populations, the incidence is higher in patients with hemodynamic instability. In a sub-analysis of the CULPRIT-SHOCK trial, new-onset AF was documented in 52 of 142 (37%) patients with cardiogenic shock complicating AMI during their index hospital stay (15). It was also observed that the majority of new-onset AF occurs in the early stages following AMI. In a community-based study, about 30% of AF occurred at the time of or within 2 days after AMI and 16% in the following 3 to 30 days (5). In our cohort, we also found that most of the new-onset AF was documented within the first 3 days of the index hospitalization. This may be partially explained by myocardial stunning and hemodynamic instability during the acute phase.

The onset of AF is dependent on triggers, perpetuators, and their interactions with the underlying substrate. As already known, older age and larger LAd are well-established risk factors of AF in the general population (16). Older age and left atrial enlargement are both related to atrial fibrosis, serving as substrates and perpetuating factors for AF, thus increasing the susceptibility of AF in AMI patients (17). Higher levels of NT-proBNP_max_ and impaired LVEF are reflective of left ventricular dysfunction and unstable hemodynamics, which indirectly increases left atrial wall tension and induces AF occurrence (18). Further, higher TnI_max_ levels could indicate a larger size of myocardial infarction, which in turn can aggravate cardiac dysfunction. Higher hCRP levels are suggestive of a more intense pro-inflammatory process. The lower levels of TSH could be a negative feedback regulation of elevated thyroid hormones caused by stress. All these excessive stress reactions in the acute phase of myocardial infarction may be the trigger for new-onset AF (19).

The angiographic data demonstrated differences in the stenosis of the LM, LAD, and LCX between the two groups, but no significant difference was found in RCA stenosis. This may be explained by whether or not the atrial branch is affected. Left atrial infarction caused by atrial branch occlusion is an independent determinant of new AF after myocardial infarction (20). Our study also identified primary PCI as an independent risk factor for new-onset AF following AMI. This may be because patients in this subgroup are more likely to develop hemodynamic instabilities which can result in the aforementioned processes. It may also be a form of reperfusion arrhythmia (21). All these new-onset AF risk factor analyses can be useful for determining which patients should be considered for extensive heart rhythm monitoring after AMI for detection of new-onset AF.

Previous studies about the impact of AF on prognosis in patients with AMI have conflicting results, with some studies showing no adverse effect (15) and others reporting an increased risk of MACE (3, 5). In this study, we separately analyzed the short-term and long-term prognostic implications of new-onset AF in AMI patients and found that the association varies between in-hospital and long-term outcomes. Our study demonstrates that new-onset AF was associated with an increased risk of in-hospital adverse outcomes even after adjustment for relevant confounders. The explanation for this may be that patients with new-onset AF were relatively older and had poorer cardiac function in the acute phase of myocardial infarction. These characteristics are not just risk factors for AF but also for adverse clinical prognosis. New-onset AF may be a marker of the clinical severity of the patients. Second, new-onset AF may lead to adverse outcomes in AMI patients through adverse hemodynamic effects caused by loss of atrial contraction, atrioventricular dyssynchrony, and rapid ventricular rates. Also, (over)anticoagulation increases the risk of bleeding, while inadequate anticoagulation increases the risk of embolism (11). No matter how carefully we evaluate and weigh the pros and cons, there remain risks. Thus, whether new-onset AF is a risk marker or causal mediator, it should not be perceived as a benign event anymore. Careful attention should be paid to patients with new-onset AF complicating AMI when making clinical decisions (22).

In patients surviving hospitalization for AMI, new-onset AF seems to have no significant association with long-term mortality in our study. AF itself can increase the risk of death (16) but we speculate that our result may be related to the lack of systematic evaluation of AF burden and compliant treatment (e.g., anticoagulation) during follow-up. We believe that transient AF episodes may not have long-term influence. Atrial fibrillation with standard treatment may not cause significant harm. Implantable monitors may be considered to detect AF recurrence in high-risk patients with new-onset AF who convert to sinus rhythm before hospital discharge (23). However, further studies are warranted to determine how transient and recurrent AF impact on long-term mortality, and to determine whether the routine use of long-term oral anticoagulation in patients with in-hospital new-onset AF improves long-term prognosis.

## Limitations

Although this is a large prospective cohort study conducted in a hospital-based setting, closer to real-world conditions, several limitations should be considered when interpreting the results. With the heart rhythm monitoring strategy used in our study, asymptomatic patients with undiagnosed AF may have been missed in the process of inclusion and exclusion; after being transferred from CCU to the general ward, intermittent ECG examinations were carried out on patients and this may also have missed transient silent AF episodes. However, this was shared by all studies addressing this topic. In this study, some patients did not undergo coronary angiography, so Takotsubo syndrome could not be completely excluded. It is reported that short- as well as long-term mortality were significantly higher in Takotsubo syndrome patients suffering from AF compared with patients without AF (24). However, all the patients received at least once echocardiography examination during the index hospitalization, which can reduce misdiagnosis rate to a certain extent. The treatment strategy utilized including rhythm versus rate control therapy during the index hospitalization, and medications during follow-up were not systematically collected in the present study, and thus, we could not make adjustments for their effects on clinical outcomes. As an observational study, we can only evaluate the associations, not capable to explain the causality.

## Conclusion

New-onset AF occurring concurrently with or after AMI during the index hospitalization is strongly associated with an increased risk of adverse in-hospital prognosis, but it does not affect prognosis in those who survive until hospital discharge. Patients with older age, enlarged left atrial, impaired cardiac function, and early reperfusion were at higher risk. Extensive heart rhythm monitoring and aggressive treatment strategies to control AF episodes should be considered to improve in-hospital prognosis in such patients.

## Data availability statement

The raw/processed data required to reproduce these findings cannot be shared at this time as the data also forms part of an ongoing study. Requests to access the datasets should be directed to HW-L, lhw19656@sina.com.

## Ethics statement

The data collection process was approved by the Institutional Review Board of Beijing Friendship Hospital affiliated to Capital Medical University and was in accordance with the Declaration of Helsinki. Written informed consent was not required for this study in accordance with the local legislation and institutional requirements.

## Author contributions

W-YY, GL, and H-WL contributed to the conception and design of the study. H-WL organized the database. W-YY performed the statistical analysis. W-YY and AF wrote the first draft of the manuscript. Z-JS, HP, W-YY, AF, and H-WL wrote sections of the manuscript. All authors contributed to the manuscript revision, read, and approved the submitted version.
